# Evaluation of Individualized Sequential Therapy Centered on Surgical Excision and Superficial Radiotherapy in Treating Keloids in the Anterior Chest Wall

**DOI:** 10.1111/jocd.71222

**Published:** 2026-10-12

**Authors:** Ming‐Li Zou, Fang‐Cheng Shen, Qian Wang, Zi‐Yu Jiang, Zhu Zhu, Hai‐Yan Zhong, Si‐Ming Yuan

**Affiliations:** ^1^ Department of Plastic Surgery, Jinling Clinical Medical College Nanjing University of Chinese Medicine Nanjing China; ^2^ Jinling Hospital, Affiliated Hospital of Medical School Nanjing University Nanjing China; ^3^ Department of Plastic Surgery, The First School of Clinical Medicine Southern Medical University Nanjing China; ^4^ Department of Plastic Surgery, Jinling Clinical Medical College Nanjing Medical University Nanjing China; ^5^ Department of Plastic Surgery Southeast University School of Medicine Nanjing China

**Keywords:** adjuvant therapy, keloids, radiotherapy, surgical treatment

## Abstract

**Background:**

This study aims to evaluate the efficacy of individualized sequential therapy centered on surgical excision and superficial radiotherapy for the treatment of sternal keloids.

**Methods:**

This study retrospectively analyzed the clinical data of 42 patients with sternal keloids admitted to a tertiary hospital in eastern China between January 2022 and December 2024. All patients received an individualized sequential therapy. Preoperatively, surgical approaches were selected based on the size and morphology of the keloids, including direct excision with tension‐reduction sutures, flap reconstruction following excision, punch excision, and a combination of surgical excision and punch excision. Postoperative sequential adjuvant therapies included superficial radiotherapy, topical anti‐scarring medications and dressings, and regular fractional laser treatment combined with compound betamethasone importation, sustained for 6 months. The Vancouver Scar Scale (VSS) and the Patient and Observer Scar Assessment Scale (POSAS) were used to evaluate scar improvement and clinical efficacy before treatment and at 12 months postoperatively. The response rate, recurrence rate, adverse reactions, and long‐term outcomes were statistically analyzed.

**Results:**

At 1 year postoperatively, VSS, OSAS, and PSAS scores decreased across all four subgroups compared with baseline. Significant differences between pre‐ and post‐treatment scores were observed in the tension‐reducing suture repair group (*n* = 18), IMAP flap reconstruction group (*n* = 9), and punch excision therapy group (*n* = 10) (all *p* < 0.05). The combined excision group (*n* = 5) showed a downward trend in postoperative scores, but the difference did not reach statistical significance (*p* = 0.063). The overall response rate was 88.1%. Postoperative radiation dermatitis occurred in 3 cases, which healed well after symptomatic management. During the follow‐up period, recurrence was observed in 5 patients (11.9%), whose symptoms were alleviated following local injections of compound betamethasone. At the end of the follow‐up period ranging from 12 to 24 months (mean: 19 months), all patients maintained stable or improved scar conditions, indicating favorable long‐term efficacy.

**Conclusion:**

Individualized sequential therapy centered on surgical excision and superficial radiotherapy, combined with other long‐term adjuvant treatments, demonstrates superior efficacy in the treatment of sternal keloids and is recommended for clinical application.

## 1 Introduction

Keloids are the result of pathological proliferation of fibrous tissue following skin injury, exhibiting infiltrative growth and local invasive characteristics, and are regarded as a quasi‐neoplastic disease [1]. Any event capable of causing dermal damage, including trauma, infection, insect bites, and burns, can induce keloid formation. The anterior chest region, characterized by high mechanical tension and hypoxic inflammation, represents the site with the highest incidence of keloids. Keloids not only cause somatic symptoms such as pruritus and pain but are also frequently associated with psychological comorbidities.

The treatment of keloids primarily relies on surgical excision combined with other adjuvant therapies. The recurrence rate following simple surgical excision of keloids ranges from 45% to 100% [2]. The anterior chest region, characterized by high tension, poor blood supply, and slower incision healing, has become the site with the highest postoperative recurrence rate of keloids [3]. Various adjuvant therapies, such as postoperative radiotherapy, topical anti‐scar ointments and dressings, laser therapy, and glucocorticoid injections, are mostly administered based on clinicians' experience, and a standardized treatment pathway has yet to be established. The individualized sequential therapy employed in this study, tailored according to scar location, morphology, size, and local skin conditions of the patient, utilizes multiple surgical modalities to repair keloid excision wounds and tension‐reducing sutures. Standardized long‐term adjuvant treatments were subsequently administered, including postoperative radiotherapy, application of external tension‐reducing devices, topical silicone gel combined with silicone sheeting, and fractional laser combined with compound betamethasone delivery. The treatment course spanned 6 months, with follow‐up conducted at 12–24 months postoperatively. The results demonstrated an overall efficacy rate of 88.1% and a recurrence rate of merely 11.9%, yielding satisfactory outcomes. The details are reported as follows.

## 2 Materials and Methods

### 2.1 Patients and Study Design

A retrospective analysis was conducted on the clinical data of 42 patients who underwent keloid treatment in the anterior chest region at a tertiary hospital in eastern China from January 2022 to December 2024. The lesions were distributed in the anterior chest wall area, bounded superiorly by the suprasternal notch and clavicular level, inferiorly by the xiphoid process and costal arch level, and laterally by the anterior axillary line. All patients provided informed consent prior to treatment and were fully informed of the potential benefits and risks. This study protocol was approved by the institutional ethics committee and was conducted in accordance with the principles of the Declaration of Helsinki.

The inclusion criteria were as follows: ① Age 18–65 years with good overall health status; ② Meeting the diagnostic criteria for keloids as defined in the Chinese Clinical Treatment Recommendation Guidelines for Keloids; ③ No prior history of radiotherapy.

The exclusion criteria were as follows: ① Presence of severe immune system diseases or insufficiency of vital organ function; ② Women who were pregnant or lactating; ③ Local infection or ulceration of the lesion; ④ History of allergy to medications used in the treatment; ⑤ Prior history of radiation dermatitis, cutaneous malignancy, or contraindications to radiotherapy; ⑥ Patients who voluntarily discharged themselves, discontinued, or abandoned treatment due to personal reasons during the course of therapy.

### 2.2 Treatment Methods

All patients underwent surgical excision of keloids with tension‐reducing sutures, followed by standardized long‐term adjuvant therapy, including postoperative superficial radiotherapy, application of external tension‐reducing devices, topical silicone gel combined with silicone sheeting, and monthly fractional laser combined with compound betamethasone delivery for 6 consecutive sessions. Comprehensive clinical efficacy evaluations were performed monthly during the first 12 months postoperatively. To enhance diagnostic and therapeutic efficiency, postoperative follow‐up visits were synchronized with laser and injection treatments. Comprehensive clinical efficacy evaluations were conducted every 6 months from 12 to 24 months postoperatively.

Surgical modality selection: (1) For multiple, scattered, and independent small‐area keloids, punch columnar excision was performed. A punch with a diameter of 1.5–3.0 mm was used to excise the keloid tissue perpendicularly, penetrating through the keloid to reach the fat layer, with a drilling interval of 1.5–3 mm. (2) For cord‐like keloids with a width of 1.5–2 cm or less, in whom preoperative pinch testing estimated that the excised scar could be approximated by primary closure, single complete excision was performed with subcutaneous tension‐reducing sutures and intradermal barbed sutures. (3) For larger keloids exceeding 2 cm in diameter, repair was performed using an internal thoracic artery intercostal perforator flap. Preoperatively, the perforator artery emergence point was mapped using a handheld doppler probe, and a propeller flap was designed. Following lesion excision, the flap was transposed to repair the wound, and tension‐reducing sutures were applied to both donor and recipient site incisions. (4) For keloids with extremely irregular morphology and extensive involvement, composite excision was employed, wherein the core area with the most significant tension was completely excised, concurrently supplemented by punch excision in the peripheral scattered regions.

Postoperative adjuvant therapy: (1) Superficial radiotherapy: Precise superficial radiotherapy was administered using an Elekta linear accelerator within 24 h postoperatively. Custom lead blocks were fabricated according to the shape of the postoperative incision, with the irradiation field encompassing the surgical incision and a 5 mm surrounding margin. A hypofractionated low‐total‐dose regimen was employed: A total of 3–4 radiotherapy sessions were delivered, with a cumulative dose of 15–20 Gy. Treatment was administered using a 6‐MeV electron beam with a 10‐mm tissue compensator applied over the postoperative incision. Leveraging the limited tissue range and rapid dose falloff characteristics of 6‐MeV electrons, this configuration concentrates the high‐dose region primarily within the reticular dermis and superficial fascia layer, while significantly reducing the radiation dose to deep chest wall structures—including the myocardium, lung tissue, and internal mammary lymphatic chains—to a clinically acceptable level. This ensures adequate therapeutic dose delivery to the target volume while minimizing radiation exposure to deep‐seated organs. (2) Sustained tension reduction: Tension‐reducing sutures were performed intraoperatively. Following the completion of postoperative radiotherapy, skin tension‐reducing devices were applied to both sides of the incision, replaced every 2–3 weeks, with a duration of no less than 6 months. (3) Topical silicone therapy: Two weeks postoperatively, once the incision had completely healed, topical silicone gel or silicone sheeting was initiated, applied for 20–24 h daily, with a duration of no less than 6 months. (4) Ablative CO_2_ fractional laser combined with drug delivery: Starting at 1 month postoperatively, fractional laser combined with Diprospan delivery was performed using the Changchun Keying CO_2_ laser system. Parameters were set as follows: single‐pulse energy 30–50 mJ (dynamically adjusted according to scar hardness), peak power 30–50 W, and fractional coverage 10%–20%. The treatment endpoint was frost‐white micropores with pinpoint bleeding. Following treatment, compound betamethasone injection (5 mg/2 mg/mL) was applied externally. For locally indurated nodules, intralesional injection of compound betamethasone was supplemented, with a single injection dose of 0.1–0.2 mL/cm^2^ according to scar area and thickness, and a total treatment volume controlled between 0.5–2.0 mL, with slight blanching of the scar tissue as the appropriate endpoint. Treatment was administered once monthly during the first 6 months postoperatively. After 6 months, the treatment interval was adjusted according to scar conditions and could be appropriately extended.

### 2.3 Evaluation of Treatment Response

#### 2.3.1 Evaluation of Clinical Efficacy

This study referenced the Darzi criteria for keloid efficacy assessment [4], combined with subjective symptoms and objective improvements in lesion appearance and texture for outcome evaluation. The categories were defined as follows: cured, markedly effective, effective, and ineffective (recurrence). Efficacy was assessed at 12 months posttreatment. Cured: the scar became completely flat, with texture approximating normal skin, and pain and pruritus completely resolved. Markedly effective: scar lesion improvement rate > 70% (including reduced thickness, diminished extent, and softened texture), with essentially complete resolution of pain and pruritus. Effective: scar lesion improvement rate of 30%–70%, with significant alleviation of pain and pruritus. Ineffective: no significant improvement in subjective symptoms such as pain and pruritus, or in the texture and appearance of the original lesion (improvement rate < 30%), or patients who initially met the criteria for cured or markedly effective but experienced recurrence during the 1‐year follow‐up period. The efficacy statistical indicator was defined as: overall effective rate = (number of cured cases + number of markedly effective cases + number of effective cases)/total number of cases × 100%.

#### 2.3.2 Assessment of Objective Scar Signs

Clinical assessments were performed using VSS and POSAS prior to treatment to establish baseline data. Re‐evaluations were conducted at 12 months postoperatively to assess therapeutic efficacy.

The VSS quantifies four domains of scar characteristics: pigmentation (0–3 points), thickness (0–4 points), vascularity (0–3 points), and pliability (0–5 points). The total score ranges from 0 to 15, with higher scores indicating more severe keloid conditions.

POSAS comprises two independent subscales. The Observer Scar Assessment Scale (OSAS) is completed by medical staff and evaluates objective scar signs based on visual and tactile assessment, encompassing six items: vascularity, pigmentation, surface roughness, thickness, pliability, and surface area. The Patient Scar Assessment Scale (PSAS) is completed by the patient and assesses subjective perceptions of the scar, including six items: pain, pruritus, color, thickness, pliability, and irregularity. Each item is scored on a scale of 1–10, where 1 indicates scar characteristics approximating normal skin, and higher scores denote more severe scarring. The total score for each subscale ranges from 6 to 60.

#### 2.3.3 Incidence of Adverse Events

Treatment‐related adverse events occurring within 12 months posttreatment were recorded, including: postoperative wound complications such as flap vascular compromise, incision dehiscence, infection, hematoma, and suture granuloma; radiation‐induced dermatitis including erythema, moist desquamation, and telangiectasia; and drug‐related adverse reactions such as skin atrophy and pigmentation changes caused by intralesional glucocorticoid injection.

### 2.4 Statistical Analysis

Statistical analysis was performed using SPSS 26.0 software. Continuous variables are presented as median and interquartile range [M (IQR)]. The Wilcoxon signed‐rank test was used to compare differences between preoperative and postoperative scores within each group (exact probability method was used for small samples). Categorical data, such as cure rates and satisfaction rates, are presented as frequency (%) and were analyzed using McNemar's test. A *p* value < 0.05 was considered statistically significant.

## 3 Results

### 3.1 Baseline Characteristics

This retrospective study enrolled 42 patients (19 males, 23 females), aged 18 to 65 years, with a mean age of 36.93 ± 15.71 years. The etiologies of keloid formation included trauma (11.9%), skin and soft tissue infection (30.95%), burns (16.67%), surgical incision (23.81%), and no apparent predisposing factor (16.67%), as shown in Table 1. Among these patients, 32 presented with a single lesion and 10 with multiple lesions. All keloids were located in the anterior chest wall region, defined as: superiorly by a horizontal line connecting the inferior borders of both clavicles at the level of the suprasternal notch, inferiorly by a horizontal line connecting the inferior borders of both costal arches at the level of the xiphoid process, and laterally by both anterior axillary lines.

**TABLE 1:** General characteristics of keloid patients.

Characteristic	Value
Sex, *n* (%)	
Male	19 (45.23)
Female	23 (54.76)
Age (years)	36.93 ± 15.71
Surgical technique, *n* (%)	
Punch excision therapy	10 (23.81)
IMAP flap reconstruction	9 (21.43)
Tension‐reducing suture repair	18 (42.86)
Combined excision	5 (11.9)
Causes of keloid formation, *n* (%)	
Accidental trauma	5 (11.9)
Soft tissue infection	13 (30.95)
Burn injury	7 (16.67)
Surgery	10 (23.81)
No obvious predisposing factor	7 (16.67)

### 3.2 Clinical Efficacy

The overall effective rate after treatment was 88.1%. According to the Darzi criteria for keloid efficacy assessment, the outcomes were as follows: 17 cases were cured (40.48%), 13 cases were markedly effective (30.95%), 7 cases were effective (16.67%), and 5 cases were ineffective (11.9%).

### 3.3 Vancouver Scar Scale (VSS)

At 1 year postoperatively, the VSS scores decreased in all four subgroups compared with baseline (Table 2). In the tension‐reducing suture repair group, the score decreased from 7.5 [7.0, 8.0] to 4.0 [3.0, 4.0] (*p* < 0.001); in the IMAP flap reconstruction group, from 10.0 [9.0, 10.5] to 4.0 [4.0, 4.5] (*p* = 0.004); and in the punch excision therapy group, from 8.0 [7.0, 8.3] to 4.0 [3.8, 4.0] (*p* = 0.002). The combined excision group showed a decrease from 9.0 [8.5, 9.0] to 4.0 [4.0, 4.0], but the difference did not reach statistical significance (exact *p* = 0.063).

**TABLE 2:** Comparison of scar scores before and after treatment among four surgical subgroups in keloid patients.

		Tension‐reducing suture repair (*n* = 18)	IMAP flap reconstruction (*n* = 9)	Punch excision therapy (*n* = 10)	Combined excision (*n* = 5)
VSS	pretreatment	7.5 [7.0,8.0]	10.0 [9.0,10.5]	8.0 [7.0,8.3]	9.0 [8.5,9.0]
	posttreatment	4.0 [3.0,4.0]	4.0 [4.0,4.5]	4.0 [3.8,4.0]	4.0 [4.0,4.0]
	*p*	< 0.001	0.004	0.002	0.063
OSAS	pretreatment	34.5 [32.8,36.0]	38.0 [36.5,40.0]	35.0 [33.0,37.8]	33.0 [31.0,33.0]
	posttreatment	11.0 [10.0,13.0]	14.0 [12.0,14.0]	15.5 [14.3,16.3]	13.0 [12.0,15.0]
	*p*	< 0.001	0.004	0.002	0.063
PSAS	pretreatment	43.5 [40.8,45.5]	45.0 [41.5,46.0]	42.5 [39.8,44.0]	42.0 [40.5,44.5]
	posttreatment	12.0 [10.0,14,0]	11.0 [10.5,14.0]	13.0 [10.0,14,0]	12.0 [10.5,14.0]
	*p*	< 0.001	0.004	0.002	0.063

### 3.4 Patient and Observer Scar Assessment Scale (POSAS)

At 1 year postoperatively, the changes in OSAS and PSAS scores among the subgroups are summarized in Table 2.

For the OSAS scores, the median values decreased from 34.5 [32.8, 36.0] to 11.0 [10.0, 13.0] in the tension‐reducing suture repair group (*p* < 0.001), from 38.0 [36.5, 40.0] to 14.0 [12.0, 14.0] in the flap reconstruction group (*p* = 0.004), and from 35.0 [33.0, 37.8] to 15.5 [14.3, 16.3] in the punch excision group (*p* = 0.002). In the combined excision group, the score decreased from 33.0 [31.0, 33.0] to 13.0 [12.0, 15.0]; however, this difference did not reach statistical significance (exact *p* = 0.063).

Similarly, for the PSAS scores, the median values decreased from 43.5 [40.8, 45.5] to 12.0 [10.0, 14.0] in the tension‐reducing suture repair group (*p* < 0.001), from 45.0 [41.5, 46.0] to 11.0 [10.5, 14.0] in the flap reconstruction group (*p* = 0.004), and from 42.5 [39.8, 44.0] to 13.0 [10.0, 14.0] in the punch excision group (*p* = 0.002). In the combined excision group, the score decreased from 42.0 [40.5, 44.5] to 12.0 [10.5, 14.0], but the difference was not statistically significant (exact *p* = 0.063).

### 3.5 Treatment‐Related Adverse Events

After treatment, 3 patients developed radiation dermatitis, which healed with delayed wound healing after dressing changes. Five patients experienced varying degrees of recurrence, which were effectively controlled after intralesional injection of compound betamethasone.

### 3.6 Representative Cases of Changes Before and After Intervention in All Groups

**FIGURE 1:**
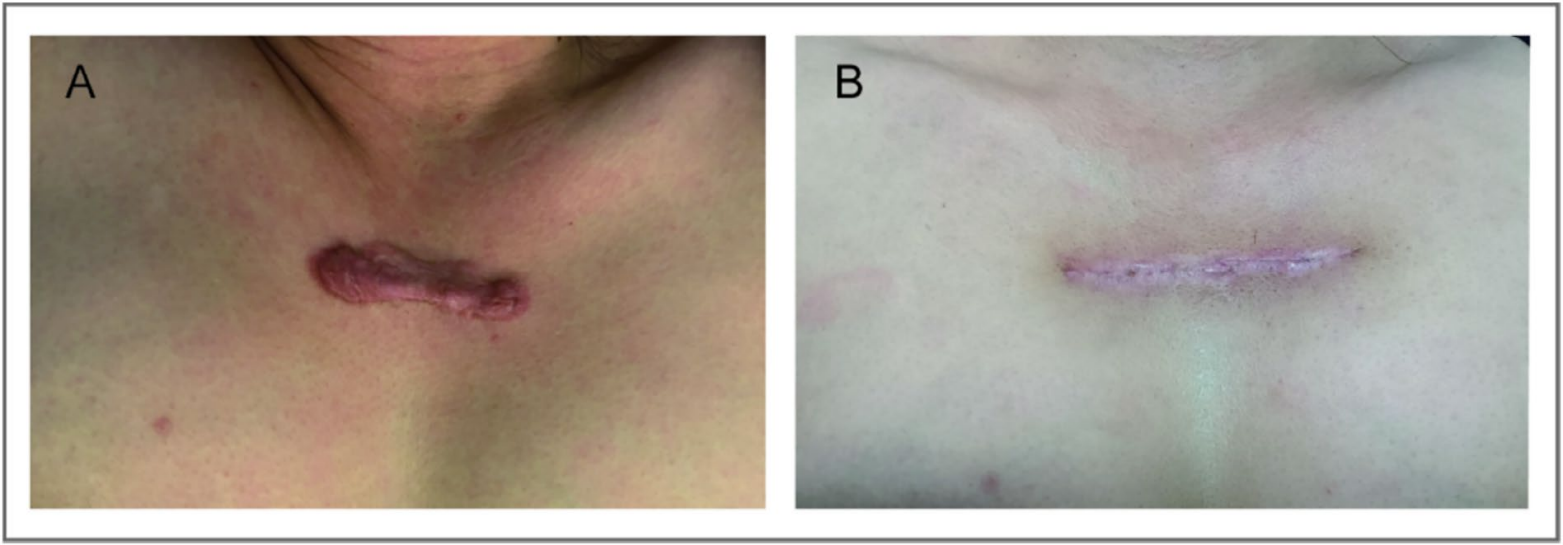
Typical Case 1: Linear keloid scar on the anterior chest (A). The patient underwent surgical excision with tension‐reducing sutures, followed by postoperative sequential adjuvant anti‐scar therapy. Efficacy assessment at 12 months postoperatively showed markedly effective outcome (B).

**FIGURE 2:**
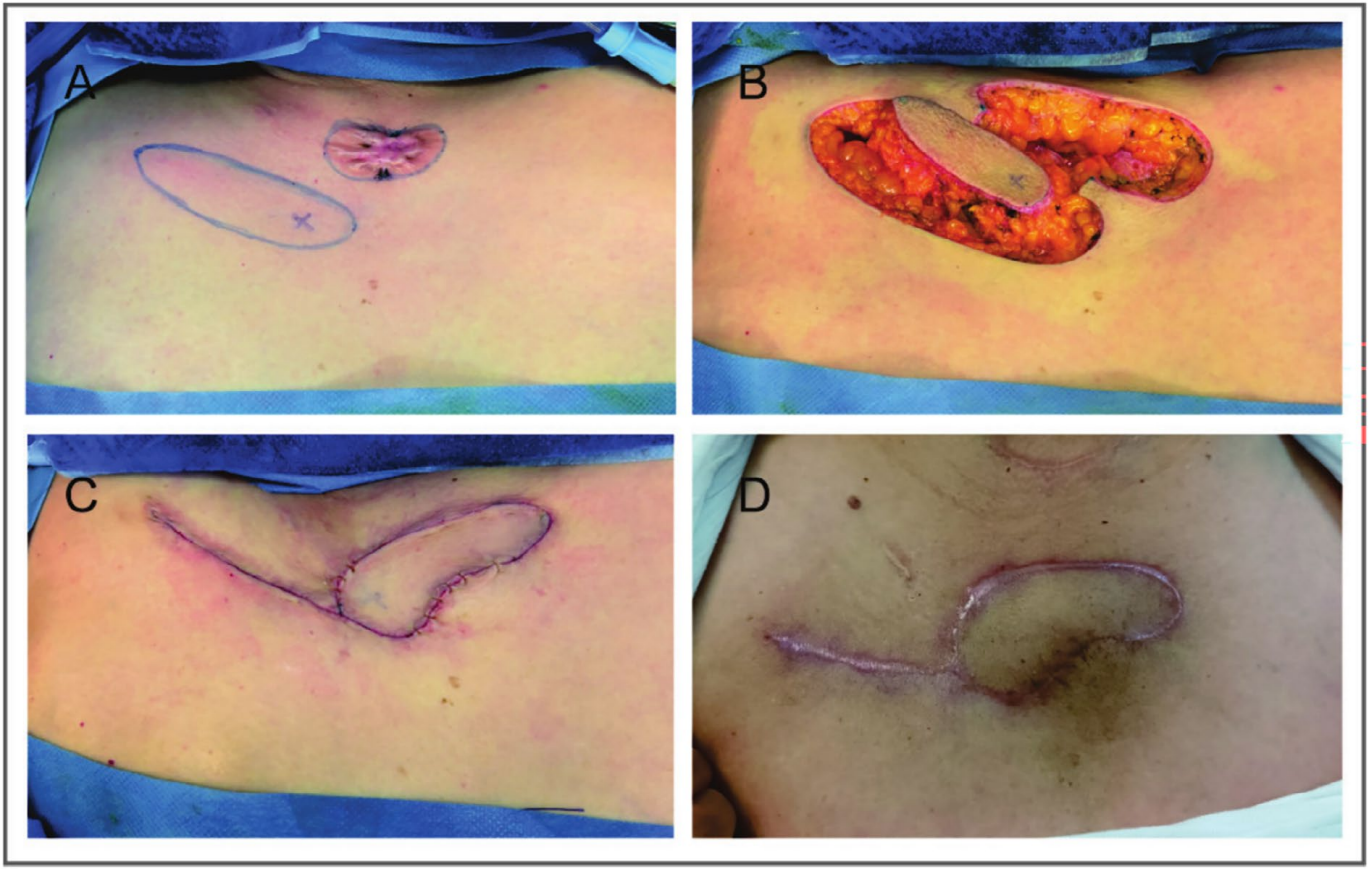
Typical Case 2: Irregular keloid on the anterior chest (A). The patient underwent surgical excision with reconstruction using an internal thoracic artery second intercostal perforator flap (B, C), followed by postoperative sequential adjuvant anti‐scar therapy. Efficacy assessment at 12 months postoperatively showed a markedly effective outcome (D).

**FIGURE 3:**
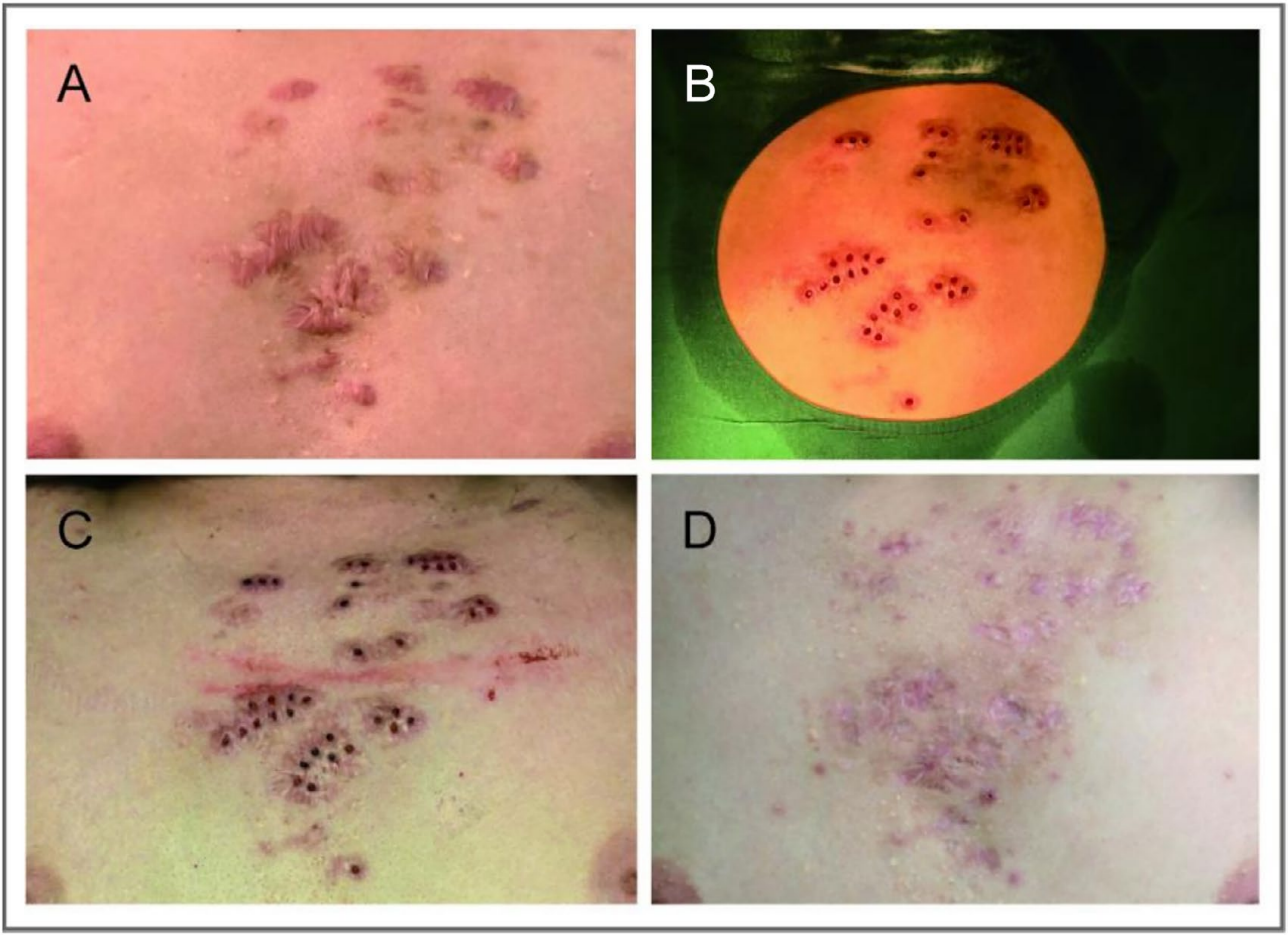
Typical Case 3: Multiple keloids of varying sizes on the anterior chest (A). The patient underwent punch columnar excision (B), followed by postoperative sequential adjuvant anti‐scar therapy (C). Efficacy assessment at 12 months postoperatively showed a markedly effective outcome (D).

**FIGURE 4:**
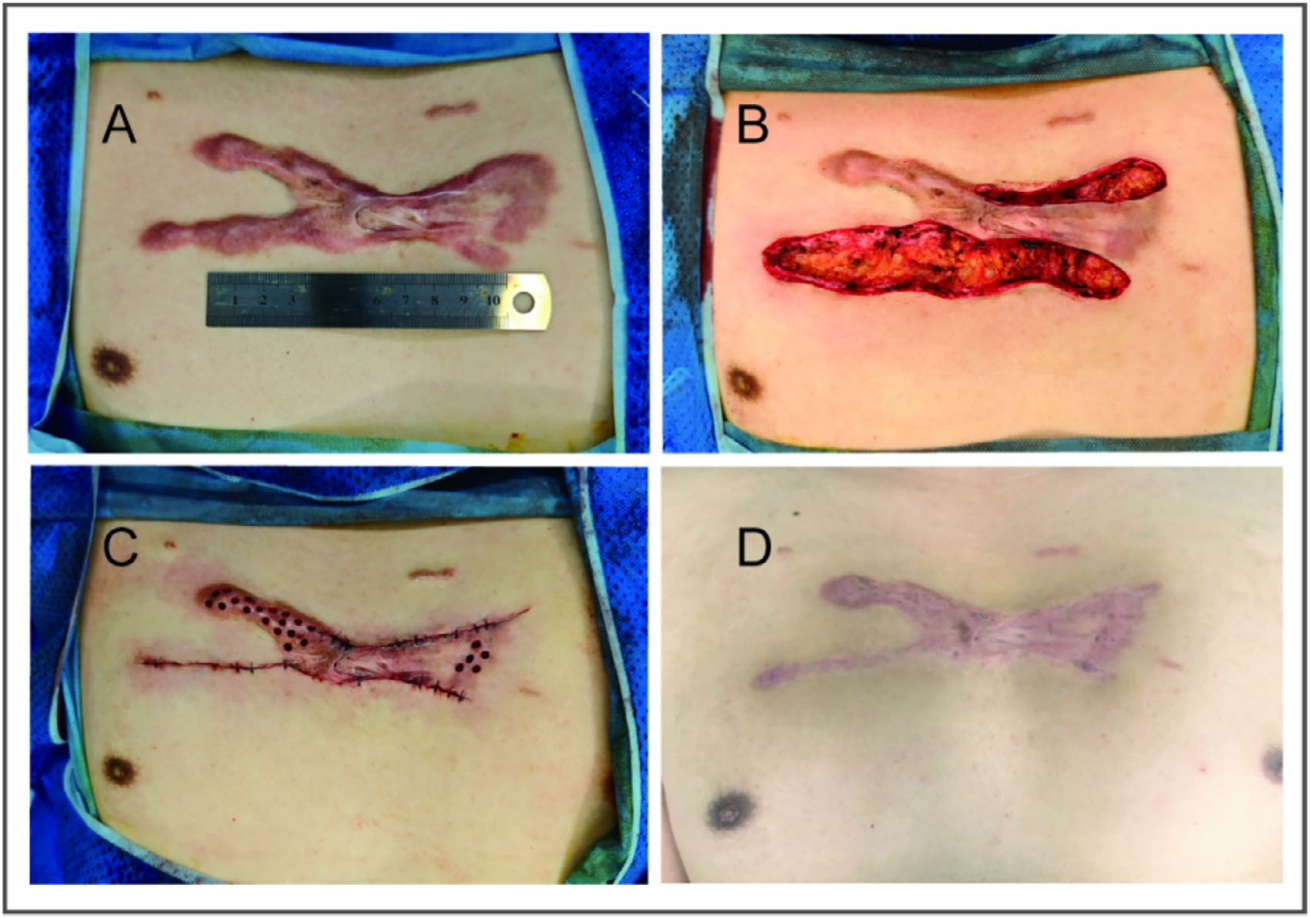
Typical Case 4: Irregular keloid scar on the anterior chest (A). The patient underwent surgical excision combined with punch columnar excision (B, C), followed by postoperative sequential anti‐scar therapy. Efficacy assessment at 12 months postoperatively showed a markedly effective outcome (D).

## 4 Discussion

The formation of keloids in the anterior chest region is attributed to the high‐tension characteristics of the skin in this area, the abundant distribution of pilosebaceous units, and the traction effects caused by continuous cardiac and respiratory activities. These factors predispose the healing process of minor injuries—such as acne, folliculitis, and surgical incisions—to become dysregulated, thereby triggering excessive fibroblast proliferation and abnormal massive collagen deposition, ultimately resulting in the formation of tough tissue extending beyond the boundaries of the initial injury [5]. This dysregulated repair phenomenon represents the concrete manifestation of an imbalance between tension and healing.; the essence of treatment lies not in combating the scar itself, but in reconstructing the intrinsic rhythm and order within the skin—namely, restoring the dynamic equilibrium between collagen synthesis and degradation, returning tension distribution to a physiologically normal state, and allowing time to truly serve as a facilitating factor for healing rather than a synergistic factor for scar formation. Accordingly, the individualized sequential therapy proposed in this study is grounded in a core logic of implementing staged, multi‐target interventions to achieve sustained scar management.

The morphology of anterior chest keloids is highly variable, necessitating individualized approaches to excision and repair. For scattered and independent keloids, the therapeutic goals are to maximally preserve normal skin bridges, minimize wound area, and reduce secondary tension. Punch excision offers unique advantages in such cases; by performing staged, partial removal of the core lesion, it reduces the disease burden, while the residual small circular wounds heal by secondary intention (Figure 3). Combined with early postoperative intervention, this approach effectively controls recurrence [6]. Park et al. [7] recently reported the “triple combination therapy,” which further validated the efficacy of 2‐mm punch partial excision combined with single‐fraction electron beam radiotherapy for multifocal trunk keloids, pioneering a minimally invasive debulking‐plus‐radiotherapy pathway. However, anterior chest wall keloids, characterized by high tension and stringent aesthetic demands, often require strategies beyond simple debulking to achieve both functional recovery and aesthetic flatness. The present study retains the punch minimally invasive strategy while incorporating complete excision combined with flap reconstruction into an individualized decision‐making system, thereby covering the full clinical spectrum from scattered small lesions to extensive complex lesions. For clustered keloids deemed amenable to single‐stage complete excision, the surgical plan is primarily determined by the morphology and size of the post‐excision defect. When the keloid is cord‐like and less than 2 cm in width, this typically indicates that longitudinal tension is the predominant issue. Following thorough lesion excision and adequate undermining of the surrounding subcutaneous tissue, reliable deep tension‐reducing suture techniques often enable direct, tension‐free closure of the wound margins [8] (Figure 1). If the keloid is mass‐like or results in a relatively large, irregular defect after excision, direct closure would generate intolerable tension, necessitating flap transfer for reconstruction (Figure 2). The selection of a flap requires comprehensive consideration of vascular reliability, arc of rotation, and impact on the donor site. The classic pedicled flap available in the anterior chest region is the internal thoracic artery perforator flap, which offers stable blood supply and is suitable for repairing wounds in the anterior chest area [9, 10]. The latissimus dorsi musculocutaneous flap or its perforator flap variants provide abundant tissue volume and extensive coverage, making them applicable for large‐area defect reconstruction in the lateral chest and breast regions [11]. The application of flaps not only achieves tension‐free coverage of the wound but also improves the local healing environment by introducing new blood supply. For keloids with extremely irregular morphology and extensive involvement, composite excision may be employed. This entails thorough excision of the core area with the most significant tension, concurrently or in stages combined with punch excision in the peripheral scattered regions (Figure 4). This strategy balances addressing the primary problem with achieving minimally invasive goals, optimizing morphological and functional recovery. It is worth emphasizing that although surgical intervention constitutes a critical component of comprehensive treatment, systematic adjuvant therapy is required postoperatively, encompassing tension reduction, radiotherapy, topical silicone therapy, fractional laser therapy, and drug delivery treatment.

This study systematically integrated and sequentially implemented the aforementioned adjuvant therapies. First, surgical intervention was employed to relieve the high‐tension state, with intraoperative tension‐reducing sutures and post‐radiotherapy application of external tension‐reducing devices to continuously decrease mechanical stress on both sides of the incision, thereby physically blocking the biomechanical foundation of scar proliferation. Subsequently, low‐dose superficial radiotherapy was initiated within 24 h postoperatively, precisely targeting fibroblasts at the cellular level to inhibit their abnormal proliferation and excessive collagen deposition [12]. The safety of the therapeutic dose used in this protocol for anterior chest wall keloids is supported by published literature: the 4‐MeV electron beam protocol of 18 Gy in 3 fractions reported by Ogawa et al. [13] and the anterior chest wall regimen of 20 Gy in 4 fractions recommended by the Japan Scar Workshop consensus [14] have both demonstrated favorable recurrence control and acceptable deep‐organ safety. After incision healing, topical silicone preparations were applied, which create an occlusive environment over the stratum corneum, reduce transepidermal water loss, establish a hypoxic microenvironment, and thereby suppress fibroblast activity and modulate collagen metabolism; daily application for 20–24 h maintains a stable local drug concentration [15, 16]. Monthly fractional laser therapy combined with compound betamethasone external application and intralesional injection was regularly performed postoperatively. The laser treatment utilizes the thermal effect of ablative CO_2_ fractional laser to initiate skin wound repair mechanisms and promote collagen remodeling, while its micropore effect enhances drug permeability, creating a synergistic interaction with injection therapy. Glucocorticoid delivery and injection inhibit inflammatory factor release and reduce collagen synthesis, synergistically enhancing anti‐scar effects when postoperative scar proliferation tendencies are identified [17]. The aforementioned adjuvant modalities target distinct pathological stages: tension reduction and radiotherapy block the “etiological factor,” silicone and pharmacological agents control the “process” and laser improves the “outcome”. This multi‐target, staged sequential strategy overcomes the limitations of monotherapy, achieving individualized and precision treatment for keloids. It is noteworthy that the efficacy of the individualized sequential therapy for keloids demonstrates a significant positive correlation with the quality of standardized whole‐course patient management.

The challenge of patient management for keloids lies not only in the disease's inherently refractory and recurrence‐prone biological characteristics, but also in the long‐term, complex nature of the management process, which places high demands on both patients and physicians. Patients often endure substantial psychological and financial burdens due to the protracted disease course, discomfort during treatment, and concerns regarding appearance, making it difficult to maintain satisfactory compliance. Physicians, in turn, must possess extensive clinical experience and interdisciplinary knowledge to achieve a delicate balance among multiple objectives: suppressing scar proliferation, improving appearance, controlling treatment side effects, and preventing disease recurrence, while simultaneously providing patients with continuous health education and psychological support. Based on these considerations, this study constructed a three‐dimensional support system of “human engagement + collaborative care + communication”: For follow‐up, dedicated personnel and standardized forms were employed, with telephone tracking to ensure that each treatment milestone was accompanied by personalized reminders and meticulous efficacy assessments. For communication, a “shared decision‐making” approach was adopted, explaining the treatment rationale to patients in accessible language to transform them from “passive recipients” to “active participants”. For resources, a multidisciplinary joint clinic was established, integrating plastic surgery, radiation oncology, and anesthesiology to provide patients with one‐stop scientific diagnosis and treatment combined with humanistic care.

Although this study has demonstrated the significant efficacy of the individualized sequential therapy for keloids, certain limitations remain. The current sample size is relatively small, which constrains the generalizability of the findings to some extent. At this stage, the study has focused exclusively on small‐to‐medium‐sized, irregular, or scattered keloids, and has not yet included giant keloids exceeding 300 cm^2^ in area. Consequently, reconstructive strategies such as tissue expander flaps and autologous scar skin replantation have not been applied in this study. Future research will expand the multi‐center sample size and include giant keloids and high‐risk recurrence populations. All patients in this study received a standardized multimodal sequential treatment regimen, without a control group receiving only the core protocol (excision + radiotherapy) or adjuvant measures alone. Therefore, we cannot definitively attribute the 88.1% response rate solely to surgical excision combined with superficial radiotherapy, nor can we quantify the independent contribution of silicone preparations, tension‐reducing devices, laser therapy, or corticosteroid injections to the overall outcome. However, it should be noted that in clinical practice for anterior chest wall keloids, monotherapy is associated with prohibitively high recurrence rates, and multimodal sequential therapy has become the prevailing strategy [18, 19]. The aim of this study was not to validate the efficacy of a single factor, but rather to evaluate the overall effectiveness and safety of this integrated treatment paradigm in a real‐world clinical setting. Therefore, future prospective controlled studies are warranted to determine the marginal benefit of each component.

In conclusion, this study adopted an individualized sequential therapy for keloids. By integrating precise preoperative assessment and surgical strategy selection with diversified postoperative adjuvant treatments, we optimized the therapeutic outcomes for anterior chest keloids. This approach provides a new paradigm for the standardized diagnosis and treatment of keloids. Future studies may further explore the combined application of emerging technologies based on these findings.

## Author Contributions

Si‐Ming Yuan and Hai‐Yan Zhong designed the research study and performed patient enrollment and management. Ming‐Li Zou, Wang qian, and Zi‐Yu Jiang performed the actual surgical operations and collected patients' clinical data. Fang‐Cheng Shen configured radiotherapy parameters for all patients and operated the radiotherapy equipment. Zi‐Yu Jiang and Zhu Zhu performed statistical analysis and data interpretation. Ming‐Li Zou and Fang‐Cheng Shen wrote the first draft of the manuscript. Si‐Ming Yuan revised and polished the manuscript.

## Funding

This work was supported by Medical Project of Jinling Hospital, 22JCYYYB28, 22JCYYYB37, 22LCZLXJS4.

## Consent

Written informed consent was obtained from all patients prior to their inclusion in this study. Written informed consent for the publication of photographs or other identifying information was obtained from all patients whose images or identifying details appear in this manuscript. All efforts were made to mask identifying features while maintaining the scientific integrity of the images.

## Conflicts of Interest

The authors declare no conflicts of interest.

## Data Availability

The data that support the findings of this study are available on request from the corresponding author. The data are not publicly available due to privacy or ethical restrictions.
